# A single-center observational study on long-term neurodevelopmental outcomes in children with tuberous sclerosis complex

**DOI:** 10.1186/s13023-023-02959-0

**Published:** 2023-11-09

**Authors:** D. Mammadova, J. Vecko, M. Hofmann, S. C. Schüssler, L. Deiters, A. Canda, A. K. Wieland, S. Gollwitzer, H. Hamer, Regina Trollmann

**Affiliations:** 1 Department of Pediatric and Adolescent Medicine, Pediatric Neurology, Friedrich-Alexander-Universität Erlangen-Nürnberg (FAU), Loschgestr. 15, 91054 Erlangen, Germany; 2 Department of Neurology, Epilepsy Center, Friedrich-Alexander-Universität Erlangen-Nürnberg (FAU), Erlangen, Germany; 3 Center of Rare Diseases Erlangen (ZSEER), Friedrich-Alexander-Universität Erlangen-Nürnberg (FAU), Erlangen, Germany

**Keywords:** Rare disease, Refractory epilepsy, Tuberous sclerosis complex, Cognition, Autism, Antiseizure medication

## Abstract

**Background:**

Tuberous sclerosis complex (TSC) is a rare multisystem disorder caused by mutations in the TSC1 or TSC2 gene. More than 90% of patients with TSC develop neurological and/or neuropsychiatric manifestations. The aim of the present study was to determine the developmental and cognitive long-term outcomes of pediatric TSC patients.

**Methods:**

This cross-sectional, monocenter study included pediatric TSC patients who received multidisciplinary long-term care with a last visit between 2005 and 2019. Neurological manifestations and cognitive development (BSID, K-ABC) were analyzed in relation to age and type of mutation.

**Results:**

Thirty-five patients aged 13.5 ± 7.8 years were included in the study. Diagnosis was confirmed genetically in 65.7% of patients (TSC1, 26.1%; TSC2, 65.2%; NMI, 8.7%). Mean age at diagnosis was 1.3 ± 3.5 years; 74.3% of the patients had been diagnosed within the first year of life due to seizures (62.9%) or/and cardiac rhabdomyomas (28.6%). The most common TSC manifestations included structural brain lesions (cortical tubers, 91.4%; subependymal nodules, 82.9%), epilepsy (85.7%), and cardiac rhabdomyomas (62.9%). Mean age at seizure onset was 1.5 ± 2.3 years, with onset in 80.0% of patients within the first two years of life. Infantile spasms, which were the first seizure type in 23.3% of the patients, developed earlier (0.6 ± 0.4 years) than focal seizures (1.8 ± 2.5 years). Refractory epilepsy was present in 21 (70.0%) patients, mild or severe intellectual impairment in 66.6%, and autism spectrum disorders in 11.4%. Severe cognitive impairment (33.3%) was significantly associated with epilepsy type and age at seizure onset (*p* < 0.05).

**Conclusions:**

The results emphasized the phenotypic variability of pediatric-onset TSC and the high rate of neurological and neuropsychiatric morbidity. Early-onset refractory epilepsy was associated with impaired cognitive development. Children of all ages with TSC require multidisciplinary long-term care and individual early-intervention programs.

**Supplementary Information:**

The online version contains supplementary material available at 10.1186/s13023-023-02959-0.

## Background

Tuberous sclerosis complex (TSC) is a multisystem genetic disorder with a prevalence of 1:5800 individuals and predominant manifestation in infancy or early childhood. Resulting from TSC1 and TSC2 gene mutations encoding the tumor suppressor proteins hamartin and tuberin, respectively, TSC is caused by an overexpression of the mechanistic target of rapamycin (mTOR) signaling pathway [1]. Due to its angiogenic and cytoproliferative properties, mTOR overexpression leads to uncontrolled cell proliferation and tumorigenesis [1], especially in the brain (e.g., cortical and subcortical tubers, subependymal giant cell astrocytomas [SEGAs], subependymal noduli [SEN]), kidneys (angiomyolipoma [AML]), and skin (angiofibroma).

Neurological manifestations present in about 90% of patients and include epilepsy, cognitive impairment, and psychiatric disorders (TSC-associated neuropsychiatric disorders [TANDs]), such as behavioral disorders, attention deficit hyperactivity disorder (ADHD), and autism spectrum disorders (ASDs) [2–4]. While data on the pediatric prevalence of TAND is limited, multicenter studies have reported an association between neurodevelopmental comorbidities and TSC2 gene mutation and age. Children and adolescents more frequently develop hyperactivity, impulsiveness, ASDs, and ADHD, while adults more frequently suffer from anxiety disorders, mood fluctuations, depression, obsessive–compulsive behavior, and psychosis. Sleep disorders and aggressive behavior develop independently of age. Normal intellectual development is reported in 24.3–46.9% of TSC patients [2, 4, 5].

Epilepsy affects 80–90% of individuals with TSC and mostly presents during early infancy; a pharmacorefractory course becomes apparent in more than two-thirds of patients [4, 6]. Tuber/nodule burden, early electroencephalogram characteristics in newborns and infants, infantile spasms, and TSC2 mutation have been proposed as putative early biomarkers of a pharmacorefractory course [7–9]. Novel therapeutic options for TSC-associated epilepsy provide promising alternatives for children and adults with TSC and include disease-modifying treatment with mTOR inhibitors (sirolimus, everolimus) [10, 11] and cannabidiol, which has recently been approved as an antiseizure medication (ASM) in TSC-associated refractory epilepsy [12]. In addition, a ketogenic diet, epilepsy surgery, and vagus nerve stimulation (VNS) are recommended as effective treatments [13]. Ongoing controlled clinical trials will clarify the potential role of preventive treatments with vigabatrin or mTOR inhibitors in newborns and young infants [14].

To meet the individual needs of patients with this multisystem disorder associated with complex comorbidities, continuous interdisciplinary management appropriate for the age and severity of the disease is crucial in the long-term care of patients with TSC [15, 16].

We present data from a retrospective monocenter study describing the seizure phenotype and TAND manifestations, and long-term outcomes in pediatric-onset TSC. Our results may contribute to improving understanding of the complexity of the disease course in children and adolescents and addressing possible gaps in disease management.

## Methods

### Patients

Our cross-sectional study enrolled children and adolescents with TSC who were treated regularly in the interdisciplinary outpatient center of the Division of Pediatric Neurology at the University Hospital of Erlangen and last presented between 2005 and 2019. All patients fulfilled the diagnostic criteria of TSC as outlined by the International TSC Consensus Conference [15]. The study was approved by the local ethics committee of the FAU Erlangen-Nürnberg. Clinical data were obtained from patient records.

### Neuropsychological testing procedures

Standardized neuropsychological tests (GES, BSID-II and III, K-ABC, HAWIK-IV) were performed by experienced occupational therapists (GES, BSID) or psychologists (K-ABC, HAWIK-IV). In case of repeated testing, the most recent result was considered. Cognitive ability was classified as “normal” (age-appropriate test performance) or “below average” (testing results below age-appropriate reference values, < 2SD) regardless of the type of testing procedure. Some patients were unable to participate in standardized testing due to severe intellectual disability; these patients were included in the category “below average.” Up to the age of 12 months, developmental outcome was assessed by the Griffiths Scales of Child Development (GES). In children aged 12–42 months, the Mental Bayley Scales of Infant Development (BSID-II and III) was applied [17]; from the age of 43 months, the Kaufman-Assessment Battery for Children (K-ABC) was used [18]. For K-ABC test interpretation, the theoretical model established by Cattell, Horn, and Carroll (CHC model) was applied, including the fluid-crystallized index (FCI), which is a comprehensive measure for intellectual processing and general intelligence. In some adolescents, cognitive ability was assessed using the Hamburg–Wechsler Intelligence Scale for Children IV (HAWIK-IV) [19]. The Autism Diagnostic Observation Schedule 2 (ADOS-2) was used to assess participants with ASDs [20]. The questionnaire was supported and analyzed by a trained psychologist.

### Statistical analysis

Data were analyzed using the statistical analysis program SPSS 26.0 for Windows (IBM Corp., Armonk, NY, USA) and are presented as mean ± standard deviation (SD). Differences between categorial variables were determined by Chi-square test of independence. Yates’ correction for continuity was applied. For comparison of mean values between two groups of metric data, the Mann–Whitney U test was applied. *p* values < 0.05 were considered statistically significant.

## Results

Patient characteristics (n = 35; female: n = 16, 45.7%; male: n = 19, 54.3%) at the time of the last visit are present in Table 1. The mean age was 13.5 ± 7.8 years; 74.3% of the patients were younger than 18 years of age. The most common first presenting symptom leading to further investigation was seizures in 62.9% and cardiac rhabdomyomas in 28.6% of the patients (prenatal diagnosis, N = 4). Mean age at diagnosis was 1.3 ± 3.5 years. TSC was diagnosed in 74.3% of patients in the first year of life and up to the age of 3 years in 97.1%. The most common organ manifestations were structural brain lesions (cortical tubers [91.4%] and SEN [82.9%]) and cardiac rhabdomyoma (62.9%). Table 2 presents the prevalence of organ manifestations diagnosed over time related to age group.

**Table 1:** Patient characteristics at last visit

N	35
Sex (n, %)	
Male	19 (54.3)
Female	16 (45.7)
CA (years; mean ± SD (range))	13.5 ± 7.8 (0.75–29.5)
CA < 18 years, n (%)	26 (74.3)
CA at diagnosis (years; mean ± SD (range))	1.3 ± 3.5 (0–20.5)
First presenting signs and symptoms, n (%)	
Seizures/epilepsy	22 (62.9)
Infantile spasms	7 (23.3)
Cardiac rhabdomyoma	10 (28.6)
Organ manifestations, n (%)*	
Cortical tubers	32 (91.4)
SEN	29 (82.9)
SEGA	8 (22.9)
Cardiac rhabdomyoma	22 (62.9)
Renal AML	15 (42.9)
Renal cysts	17 (48.6)
Retinal hamartoma	2 (5.7)
Epilepsy, n (%)	30 (85.7)
Genetics, n (%)	
Testing completed	23 (65.7)
TSC1 mutation**	6 (26.1)
TSC2 mutation	15 (65.2)
NMI	2 (8.7)
Positive family history for TSC	12 (34.3)

*Except skin manifestation

**In one patient without skin and organ manifestation other than FCD (focal cortical dysplasia) TSC1 mutation was found in brain tissue following epilepsy surgery

Abbreviations: CA, chronological age; NMI, no mutation identified; SEGA, subependymal giant cell astrocytoma; SEN, subependymal nodule(s); AML, angiomyolipoma

**Table 2:** Overview of organ manifestations at last visit by age group (N = 35)

Manifestation	Age group			
N (%)	0–4 years	5–12 years	13–17 years	≥ 18 years
	n = 6	n = 11	n = 8	n = 10
Cortical tubers	5 (83.3)	10 (90.9)	7 (87.5)	10 (100.0)
SEN	6 (100.0)	9 (81.8)	6 (75.0)	8 (80.0)
SEGA	0 (0.0)	4 (36.4)	2 (25.0)	2 (20.0)
Cardiac rhabdomyoma	5 (83.3)	6 (54.5)	8 (100.0)	3 (30.0)
Renal AML	1 (16.7)	5 (45.5)	5 (62.5)	4 (40.0)
Renal cysts	3 (50.0)	5 (45.5)	6 (75.0)	3 (30.0)

*SEN* Subependymal nodule(s); *SEGA* Subependymal giant cell astrocytoma; *renal AML*, Renal angiomyolipoma(s)

### Structural central nervous system anomalies

Cerebral magnetic resonance imaging (MRI) pathologies were initially detected at the mean age of 1.2 ± 1.0 years. Cortical tubers and SEN were already present in young infants, whereas SEGAs were diagnosed at the mean age of 4.9 ± 5.4 (0.5–12.0) years. SEGAs located near the foramen of Monro exhibited moderate size progression in three patients.

### Epilepsy

**Fig. 1:**
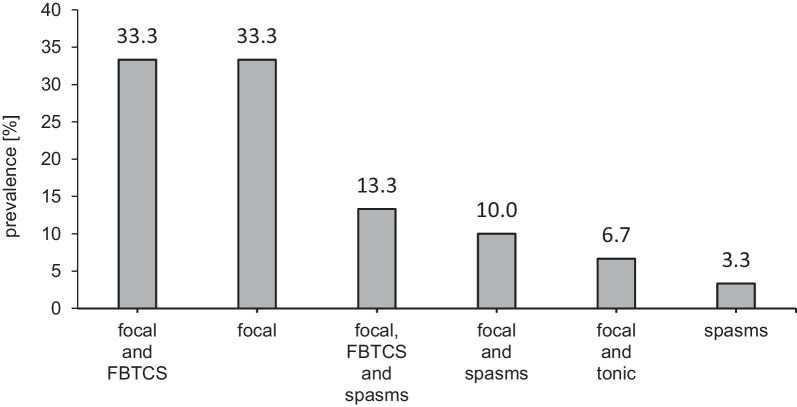
Seizure types in tuberous sclerosis complex patients with epilepsy (N = 30) up to the last visit. FBTCS, focal to bilateral tonic–clonic; ISS, infantile spasm syndrome; LGS, Lennox–Gastaut syndrome

Epilepsy was diagnosed in 85.7% (30/35) of the patients, without marked differences in prevalence between the age groups: 0–4 years, 5 (83.3%); 5–12 years, 9 (81.8%); 13–17 years, 7 (87.5%); and ≥ 18 years, 9 (90.0%). The first seizure occurred at a mean age of 1.5 ± 2.3 (0.1–10) years, with an earlier manifestation of infantile spasms than focal seizures. In 76.7% (23/30) of patients, the first seizure type was focal and occurred at a mean age of 1.8 ± 2.5 (0.1–10.0) years, while infantile spasms were documented as the first seizure type in 23.3% (7/30) of patients at a mean age of 0.6 ± 0.4 (0.1–1.5) years. First seizures occurred within the first year of life in 63.3% of patients with epilepsy and in the first two years of life in 80.0% of patients with epilepsy. An overview of seizure types is shown in Fig. 1. Twenty patients (66.7%) had exclusively focal seizures with and without focal to bilateral tonic–clonic seizures. Infantile spasms and/or Lennox–Gastaut syndrome (LGS) developed in 10/30 (33.3%) patients. Five (14.3%) patients did not develop epilepsy up to the time of the last visit (0.75, 6.4, 9.2, 15.8, and 18.9 years of age).

### Antiseizure medications

**Fig. 2:**
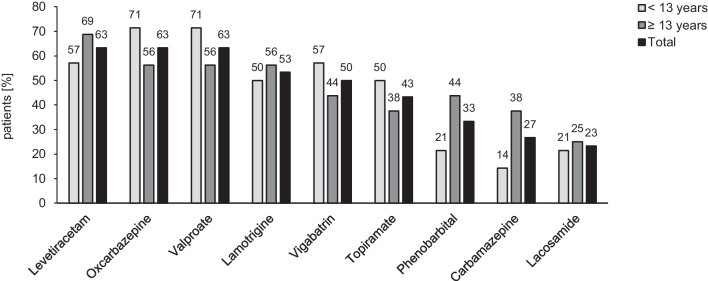
Most frequent antiseizure medications (ASMs) used in patients with epilepsy (n = 30) during the observational period by age group (< 13 years, n = 14; ≥ 13 years, n = 16)

Figure 2 summarizes the most common ASMs used in patients with epilepsy (n = 30) in relation to age group. On average, patients received 5.2 ± 3.1 (1–11) ASMs during the observational period. At the time of the last visit, the most common drugs were valproate, oxcarbazepine, lamotrigine, vigabatrin, lacosamide, and levetiracetam (Table 3). Vigabatrin and topiramate were more common in the younger age group, while lamotrigine and lacosamide were more common in patients from the age of 13 years. Twenty-one (70.0%) patients did not achieve seizure freedom. Epilepsy surgery (e.g., lesionectomy, temporal lobe standard resection) had been performed in four (13.3%) patients at the ages of 0.3, 4.0, 6.0, and 20.7 years, of whom two achieved seizure freedom. Six (17.1%) patients (mean age 11.0 ± 8.3 years; range 1.1–25.0) were treated with everolimus for 3.0 ± 2.4 (0.5–6.6) years due to refractory epilepsy, SEGA (N = 3), and/or renal AML (N = 3). In one patient, treatment was discontinued because of serious side effects (stomatitis and ulcers), and one patient developed severe anemia.

**Table 3:** Most common antiseizure medications by age group (at the time of last visit)

	< 13 years	≥ 13 years	Total
	N = 14	N = 16	N = 30
	N (%)	N (%)	N (%)
Valproate	7 (50.0)	6 (37.5)	13 (43.3)
Oxcarbazepine	7 (50.0)	4 (25.0)	11 (36.7)
Lamotrigine	3 (21.4)	5 (31.3)	8 (26.7)
Vigabatrin	4 (28.6)	3 (18.8)	7 (23.3)
Lacosamide	1 (7.1)	4 (25.0)	5 (16.7)
Levetiracetam	1 (7.1)	4 (25.0)	5 (16.7)

Others: CBZ, ESM, Perampanel, Phenobarbital, Bromide, Pregabalin, Rufinamide, Topiramate, Sulthiam, Felbamat

### Cognitive development

**Fig. 3:**
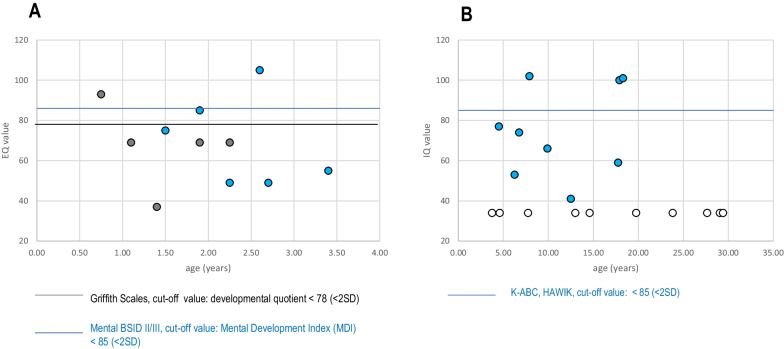
Neuropsychological functioning in patients with TSC in relation to age. **A** Patients < / = 42 months of age (N = 11), individual results of GES (black) and BSID (blue). **B** Patients > 42 months of age: individual results of K-ABC and HAWIK (blue), and patients who were unable to participate in standardized testing due to severe intellectual disability (white). n.s

Data on cognitive development were available in 33/35 patients. Standardized neuropsychological testing was performed for 20/35 (57.1%) patients. Thirteen patients with severe cognitive impairment were assessed clinically as standardized testing could not be performed. In children below the age of 42 months (n = 11), standardized testing (GES, BSID) has been performed at the mean age of 2.1 ± 0.8 (0.8–3.4) years. Individual values are given in Fig. 3. Nine patients (81.8%) had a developmental index below normal range (MDI < 85, GES score < 78). Using IQ testing (K-ABC II, HAWIK), FCI were determined in 9 patients at a mean age of 11.3 ± 5.5 (4.5–18.2) years (> 12 years of age: 4/9, 44.4%). Cognitive performance was below average in 6/9 patients. These patients showed a mean FCI of 69 (41–102; n = 6; K-ABC) or 87 (59–101; n = 3; HAWIK) (Fig. 3). Overall, including patients who were not testable, 33.3% (11/33) of patients demonstrated average performance, and 66.6% (22/33) cognitive impairment, including severe intellectual impairment in 33.3% (11/33). Table 4 demonstrates cognitive performance in relation to genotype and severity of epilepsy (N = 21; 2 patients with NMI were not included in the table). In the “average cognitive performance” group (n = 5), mean age at first seizure was 3.5 ± 4.12 (0.25–10.0) years, whereas in the “below-average cognitive performance” group (N = 12), mean age at first seizure was 0.48 ± 0.56 (0–2.0) years (*p* < 0.05). Seizure onset in patients with severe intellectual impairment occurred at a mean age of 0.19 ± 0.19 (0–0.5) years, thus, significantly earlier than in the “average cognitive performance” (Table 4, Additional file 1: Tables A1 and A2). Early-onset seizures were significantly associated with a higher risk of cognitive impairment (*p* = 0.030). Infantile spasms and LGS were more often associated with severe cognitive impairment (50.0% and 100.0%, respectively) than focal seizures (22.2%) (*p* = 0.372).

**Table 4:** Patient´s characteristics in relation to genotype and developmental outcome, n = 21 (age, mean ± SD: 7.4 ± 6.9 years)

Cognitive ability	Average	Below average (< 2SD)	Subgroup: Severe cognitive impairment
	n (%)	n (%)	n (%)
Genotype			
TSC1	2/6 (33.3)	4/6 (66.7)	2/6 (33.3)
TSC2	5/15 (33.3)	10/15 (66.7)	4/15 (26.7)
Epilepsy			
Age at manifestation (years), mean ± SD	3.45 ± 4.12	0.48 ± 0.56	0.19 ± 0.19*
No epilepsy, N	2	2	-
Epilepsy with focal seizures, N (%)	4/11 (36.3)	7/11 (63.7)	2/11 (18.2)
Multifocal epilepsy with various seizures types (incl. West syndrome, LGS), N (%)	1/6 (16.7)	5/6 (83.3)	4/6 (66.7)
Seizure freedom (last visit)			
No seizure freedom (last visit)	4/12 (33.3)	8/12 (66.7)	3/12 (25.0)
Seizure freedom > 1 year (last visit)	1/5 (20.0)	4/5 (80.0)	3/5 (60.0)

*p < 0.05

ASDs were diagnosed in 4/35 (11.4%) patients (TSC1 mutation, n = 1; TSC2 mutation, n = 2) at a mean age of 7.0 ± 2.2 (4.2–9.4) years. Speech developmental disorder was present in 24/34 (70.6%) patients; communication was restricted to a few words in 14.7% (5/34) of patients. At the last visit, 8 (23.5%) patients attended a regular school, 23 (67.6%) attended an institution for people with special needs, and 5 worked in special facilities. While 80.0% (28/35) of patients were able to walk without support, 8.6% (3/35) needed assistance.

### Genotype–phenotype correlations

Genetic testing performed in 65.7% (23/35) of the patients at a mean age of 4.6 ± 5.9 (0.0–20.0) years confirmed pathogenic variants in 21 patients in the TSC1 (N = 6, 26.1%) or TSC2 gene (N = 15, 65.2%). No mutation was identified (NMI) in 2/23 (8.7%) patients. The ratio of TSC1:TSC2 mutations was 1:2.5. As shown in Table 5, there were no significant correlations between age at manifestation or severity of organ manifestations and genotype, except a significantly higher association of SEN and renal cysts with mutations in TSC2 than in TSC1. Family history revealed 10 affected first-degree relatives and 6 s-degree relatives in 12 families (12/32, 37.5%).

**Table 5:** Genotype-phenotype correlations (N = 23)

	Parameter present			Parameter not present			*p* value*
	Genetic test available	TSC1	TSC2	Genetic test available	TSC1	TSC2	
	n	n (%)	n (%)	n	n (%)	n (%)	
Positive family history	5/12	1 (20.0)	4 (80.0)	14/23	5 (35.7)	9 (64.3)	1.000
Cortical tubers	19/32	5 (26.3)	14 (73.7)	0/3	0 (0.0)	0 (0.0)	^†^
SEN	18/29	3 (16.7)	15 (83.3)	3/6	3 (100.0)	0 (0.0)	0.015
SEGA	5/8	0 (0.0)	5 (100.0)	16/27	6 (37.5)	10 (62.5)	0.262
Epilepsy	17/30	5 (29.4)	12 (70.6)	4/5	1 (25.0)	3 (75.0)	1.000
Epilepsy type							1.000
Focal	11/20	3 (27.3)	8 (72.7)	0/15	–	–	
Infantile spasms	6/8	2 (33.3)	4 (66.7)	0/27	–	–	
LGS	0/2	–	–	0/33	–	–	
No epilepsy	4/5	1 (25.0)	3 (75.0)	0/30	–	–	
Epilepsy surgery	3/4	2 (66.7)	1 (33.3)	18/31	4 (22.2)	14 (77.8)	0.184
VNS	1/1	0 (0.0)	1 (100.0)	20/34	6 (30.0)	14 (70.0)	1.000
Cardiac rhabdomyoma	13/22	2 (15.4)	11 (84.6)	8/13	4 (50.0)	4 (50.0)	0.146
Renal AML	10/15	2 (20.0)	8 (80.0)	11/20	4 (36.4)	7 (63.6)	0.635
Renal cysts	11/17	0 (0.0)	11 (100.0)	10/18	6 (60.0)	4 (40.0)	0.004
Retinal hamartoma	1/2	0 (0.0)	1 (100.0)	20/33	6 (30.0)	14 (70.0)	1.000
Cognitive performance							1.000
Average cognitive abilities	7/11	2 (28.6)	5 (71.4)	0/24	–	–	
Below-average cognitive abilities	8/11	2 (25.0)	6 (75.0)	0/24	–	–	
Severe mental impairment	6/11	2 (33.3)	4 (66.7)	0/24	–	–	
Autism	3/4	1 (33.3)	2 (66.7)	18/31	5 (27.8)	13 (72.2)	1.000
Everolimus	4/6	0 (0.0)	4 (100.0)	17/29	6 (35.3)	11 (64.7)	0.281

TSC1 mutation was detected in 6 patients and TSC2 mutation in 15 patients

Presented are numbers of patients who underwent genetic testing

*Statistical analysis had been performed to determine correlations between genotype (TSC1 or TSC2) and phenotype (examined parameter) in consideration of the number of patients in which the examined parameter is not present. Fisher’s Exact Test was used to determine significance (*p* < 0.05)

^†^Since all patients who underwent genetic testing had cortical tubers, no statistical analysis could be performed

### Developmentally supportive care

**Fig. 4:**
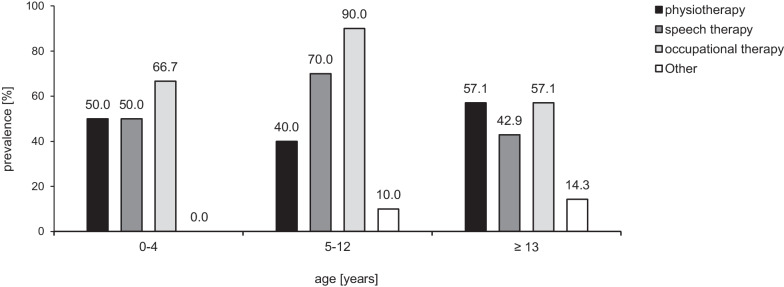
Developmental supportive care in relation to age group (N = 30)

As summarized in Fig. 4, the rate of multidisciplinary supportive care was highest in children < 13 years of age, with occupational and speech therapy in 66.7–90% and 50.0–70.0% of the patients, respectively; however, more than one-half of adolescents aged 13 years or older also used supportive care from physiotherapists and speech and occupational therapists.

## Discussion

This retrospective study found wide phenotypic variability in pediatric-onset TSC, which was more often caused by TSC2 than TSC1 gene variants. Furthermore, the results emphasized the high rate of complex neurological and neuropsychiatric morbidities from the first year of life in addition to the need for interdisciplinary long-term care. While seizures and cardiac rhabdomyomas were the most common symptoms leading to diagnosis, more than 85% of the children revealed structural brain lesions (cortical tubers, 91.4%; SEN, 82.9%), suffered from TSC-associated epilepsy with early onset during the first two years of life (80.0%), and mild/severe intellectual impairment (66.6%). Deficits in cognitive performance were significantly associated with age at seizure onset and epilepsy type (*p* < 0.05). There was no significant relationship between the severity of pediatric-onset neurological and/or multiorgan manifestations and genotype, except a significantly higher association of SEN and renal cysts with mutations in the TSC2 gene.

### Specific TSC manifestations in relation to age

The mean age at diagnosis of 1.3 ± 3.5 years in our study population was similar to the international TOSCA study, which included 2093 children and adults [4]; the mean age was slightly higher than the prospective German ESPED study (N = 86; mean age at diagnosis: 11 months) [21], which included patients under the age of 18 years (2015–2017). The most common first signs and symptoms in our patients were seizures (62.9%) and cardiac rhabdomyoma (28.6%). Prenatal suspicion of TSC due to ultrasonographic diagnosis of cardiac rhabdomyoma in 11.4% of our cohort was rare compared with prospective data from the ESPED study (22.1%) [21]; however, reports in the literature on prenatal diagnosis (5.9% [4]; 22.1% [21]) and overall prevalence of cardiac rhabdomyoma vary (34.3–59.3%) [4, 21].

Consistent with other studies [4, 21], the most common specific TSC manifestations were neurological manifestations with cortical tubers (91.4%), SEN (82.9%), and epilepsy (85.7%), followed by cardiac rhabdomyoma (62.9%) and renal manifestations (renal cysts, 48.6%; AML, 42.9%), equally affecting both sexes. Cortical tubers, SEN, and renal cysts were similarly prevalent across all age groups, whereas SEGAs and renal AMLs occurred more often in older children. This is in line with other studies reporting a peak prevalence of SEGAs and retinal hamartomas in childhood, whereas the number of renal AMLs increase through adulthood up to 83.2% [22]. In general, age-related prevalence of organ manifestations and variable methodological designs and definitions could explain the reported differences in TSC manifestations. As recommended by international experts [15, 16], these age-dependent organ manifestations and associated challenges must be considered when planning individual long-term follow-up.

### TSC manifestations by genotype

Most of our cohort had pathological TSC2 variants (65.2% of all genetically tested children), 26.1% had TSC1 variants, and the rate of NMI was 8.7%. This TSC1:TSC2 ratio of 1:2.5 is in line with previous prospective German data [21] and other studies [4]. In the German study, genetic testing performed in 53.5% of the patients detected pathological variants in TSC1 in 21.7% and variants in TSC2 in 58.7% of the patients, while the rate of NMI was 19.6% [21]. The true mutation rate of TSC1 may have remained undetected due to its milder associated phenotype [23]. In addition, with the increasing use of next generation sequencing, the rate of NMI will probably decrease in the near future [24].

The present data suggest that patients with TSC1 variants have a less severe phenotype than patients with TSC2 variants; however, due to the small cohort size, this difference did not reach statistical significance, except for SEN and renal cysts, which were significantly more often associated with TSC2 than TSC1 variants. Furthermore, SEGA, cardiac rhabdomyomas, and renal AML occurred more frequently in patients with TSC2 variants than patients with TSC1 variants. This is in accordance with other studies reporting that SENs, retinal hamartomas, and renal AMLs are more frequently found in patients with TSC2 than TSC1 mutations, while cortical tubers and hypomelanotic macules occur at similar frequencies across all patients [23]. Due to the large clinical variability—even within the same family—prediction of the course of the disease is limited [24, 25]. Salussolia et al. [25] summarized the mutational variants registered in Leiden Open Variation Database and demonstrated that, in the TSC1 gene, exon 15 was the most frequently affected region, and more than one-quarter of TSC2 mutations were located in exons 16, 23, 33, and 40. In our patients, most of the TSC2 mutations were also detected in exon 33.

### Epilepsy in relation to age and genotype

Epilepsy in TSC is associated with seizures that are refractory to ASM in about two-thirds of pediatric and adult patients, severely impairing the quality of life (QoL) of patients and their caregivers [9, 26–29]. In our patients, epilepsy was diagnosed in 85.7% at a mean age of 1.5 ± 2.3 years, and 63.3% of seizures occurred within the first year and 80.0% within the first two years of life. A similar prevalence (83.6%) was observed in the 3^rd^ interim analysis of the TOSCA study including 2216 patients [30], in a retrospective survey at the Massachusetts General Hospital (85.2%, n = 291) [26], and in the evaluation of the TSC Natural History Database (83.6%; n = 1328) [31]. The present data confirmed a high rate of infantile spasms (33.3%) and typical onset pattern of infantile spasms and focal seizures in the first two years of life [30–32]. Furthermore, infantile spasms developed markedly earlier than focal seizures in our patients (0.6 years vs. 1.8 years, respectively). This is in line with literature reporting a prevalence of infantile spasms in TSC of 34.7–38.9% [6, 26, 30, 33, 34]. Higher rates were observed in a Dutch study (44.0%) [35] and an American prospective multicenter study (57%) [32]. Recent data from TOSCA demonstrated an early onset of infantile spasms at a median age of 4 months, whereas focal seizures were diagnosed at a median age of 1 year [6]. These authors highlighted an overall improvement in the early diagnosis of infantile spasms in TSC patients over the last decade. LGS, representing a severe epileptic encephalopathy associated with a cognitive decline and pharmacorefractory epilepsy, developed in 6.7% of our patients. Similarly, in a cohort of 106 TSC patients, LGS was reported in 7.8% of patients [26]. In a pediatric TSC cohort consisting of 95 patients, LGS was diagnosed in 3.2% of the patients within 5 years after TSC diagnosis [33].

Of note, epilepsy onset occurred later in life in our patients with TSC1 variants than patients with TSC2 variants (3.2 years vs. 0.6 years, respectively). In addition, patients with TSC1 variants were more often seizure free at the time of the last visit than patients with TSC2 variants; however, this difference was not statistically significant. Previous studies similarly reported a higher frequency of epilepsy in general [32, 36], infantile spasms, and refractory focal epilepsy [26, 37] in patients with TSC2 compared with TSC1 variants.

The high burden of refractory epilepsy constitutes a significant challenge in the treatment of TSC patients. Similar to another study [35], we observed seizure freedom > 1 year in only 30.0% of the children and adolescents with TSC using standard ASMs.

### Antiseizure medications

The most used antiepileptic drugs in our patients who received 5.2 ± 3.1 ASMs during the observation period were valproate (63.3%), oxcarbazepine (63.3%), levetiracetam (63.3%), lamotrigine (53.3%), vigabatrin (50.0%), and topiramate (43.3%). This is in line with previous reports [33, 34, 38] and recent observations from a German multicenter survey in 2019 on age-stratified patterns of ASM use among children, adolescents, and adults with TSC [39]. These reports indicated that more than 60% of patients used a combinatory therapy of 2–4 ASMs (62.3%) and that the most used individual ASMs in polytherapy were valproate (45.5%), lamotrigine (44.3%), oxcarbazepine (27.4%), and levetiracetam (22.8%). Treatment with vigabatrin (21.0%) and topiramate (7.8%) was less often documented; however, this varied by age group. Vigabatrin was used in 58% of children under the age of 5 years and in 36% under the age of 10 years. Real-world data on ASM utilization in pediatric TSC patients is limited. In patients < 18 years of age, studies have reported valproate (54.7–60.0%), vigabatrin (28.4–46.0%), levetiracetam (33.0–46.3%), carbamazepine (29.0–38.9%), oxcarbazepine (16.0–37.8%), lamotrigine (23.0–37.8%), and topiramate (13.0–44.2%) as the most common ASMs [33, 35, 39], reflecting expert recommendations on TSC-associated epilepsy [13]. Valproate was used as often as oxcarbazepine and lamotrigine in our study group, probably due to the high proportion of refractory epilepsy [40]. The individual treatment benefit of valproate must be estimated and re-evaluated during the course of therapy against the known adverse side effects, including toxicity and teratogenicity. According to current treatment recommendations and considering its proposed GABAergic mode of action [13], topiramate was used as an add-on for focal seizures in 43.3% of our patients during the course of epilepsy, in a higher proportion than was observed in other studies [35, 39] but similar to Lennert et al. [33], who reported on young pediatric TSC patients from a tertiary care epilepsy center. Up to the time of the last visit, topiramate was discontinued by most of the children in our study (< 10%).

Vigabatrin is highly effective in treating infantile spasms and focal seizures in children < 1 year of age [41], and it is recommended as a first line ASM in TSC-associated infantile spasms and focal seizures with onset before the age of 1 year [13]. This may explain the high frequency of VGB use in younger patients. Overall, 15 of our patients used VGB; among them, 46.6% used VGB for the treatment of infantile spasms, which is in accordance with previous findings [30, 33]. From recent studies, evidence suggests that pre-symptomatic treatment of infants with VGB (n = 54) could prevent infantile spasms, delay epilepsy onset, and decrease the severity of epilepsy compared with treatment after the onset of clinical seizures (EPISTOP) [41]. Further long-term follow-up is needed for the assessment of cognitive outcomes and neuropsychiatric disorders.

As everolimus has been approved for the treatment of TSC-associated refractory focal epilepsy in patients with TSC aged ≥ 2 years [5] since 2017, only a limited experience was reported in our retrospective study (13.3% of patients), which included patients with a last visit in 2019. This is in line with observations on the use of mTOR inhibitors (1%) from the multicenter TSC Natural History Database, which included patients between 2006 and 2014 [31] and interim analysis of the TOSCA study in 2015 reporting everolimus treatment in 7.7% of patients with focal seizures and 5.5% with infantile spasms [30]. Use of everolimus was reported in 32.5% of patients in a 2019 German survey [39], indicating an increasing use of this disease-modifying therapeutic option among individuals with TSC. Similarly, the most recent analysis of epilepsy characteristics in the TOSCA population demonstrated this continuing increase in the use of everolimus over time, reaching 18.1% [6].

Concerning non-pharmacological treatments of refractory epilepsy, epilepsy surgery was performed in 13.3% of our patients and VNS in one child. Similar low frequencies of epilepsy surgery (6.5–8.4%) and VNS (3.8–6.0%) in TSC-associated epilepsy were reported in other studies [6, 30, 33, 38]. As epilepsy surgery in TSC is described as a promising therapeutic modality in selected patients to minimize seizure burden and neurodevelopmental consequences and to improve QoL [42–44], early pre-surgical evaluation in specified epilepsy centers should be provided to children and adults with drug resistant TSC-associated epilepsy [16]. Furthermore, VNS is established and recommended for the treatment of drug resistant TSC-associated epilepsy [45, 46]; however, randomized-controlled trials are missing. Ketogenic diets, which effectively reduced seizure frequency in a small and heterogenous study group of patients with TSC [47, 48], were not applied in our patients, which was comparable to other studies in which this treatment option was rare in TSC patients [30, 38]. One explanation could be that randomized-controlled studies on these therapeutic options are still lacking.

### Neuropsychiatric comorbidities in relation to age and genotype

We observed impaired cognitive development in 66.6% of our patients, of whom 50.0% showed severe cognitive impairment. Disorders of speech and language development were diagnosed in 70.6% of our patients, ASDs in 11.4%, and 67.6% of our patients attended institutions for people with special needs. Compared with previous studies reporting a prevalence of cognitive impairment in TSC patients between 50.8 and 60.0% [4, 49], the prevalence of cognitive impairment in our study was slightly higher. In agreement with Ruiz-Falko et al. [5], who concluded from the recently published TOSCA PASS data (data cutoff date: 22 January 2020) that monitoring and reporting of TAND in patients with TSC in the EU is widely inadequate, we suggest that variable study results on cognitive development in pediatric TSC patients may result from missing standardized data. The proportion of pediatric patients in PASS was 34.1% (n = 61) compared with 65.9% (N = 118) adults. IQ scores were available in only 31% of the whole study group (57/179). Among them, 28.1% had normal intellectual ability, while mild, moderate, severe, and profound intellectual disability was observed in 40.4%, 22.8%, 17.5% (N = 10), and 5.3% (N = 3) of patients, respectively. A markedly higher proportion of IQ scores was available in pediatric patients (41/61; 67.2%); among them, normal IQ values were reported in 10 patients (24.3%), while mild, moderate, severe, and profound intellectual disability was reported in 17 (41.4%), 17 (41.4%), 9 (21.9%), 7 (17.0%), and 3 patients (7.3%), respectively. In line with our results and others (57%) [49], the study supported a high rate of intellectual disability in the pediatric age group (75.7%).

In addition to the reported observations that phenotypes associated with TSC1 and TSC2 variants overlap substantially [35], we did not observe significant differences of cognitive ability in relation to the type of TSC mutation in our retrospective analysis. However, evidence from the literature suggests that TSC2 mutations are associated with a higher proportion of severe phenotypes, developmental deficits, and intellectual disability than TSC1 mutations [50].

Among prognostic markers for neurodevelopmental outcome in TSC patients, refractory focal epilepsy was proposed as an unfavorable factor in several studies [26, 35]. We demonstrated that an early onset of epileptic seizures before the age of 12 months was significantly associated with a poorer cognitive performance compared with later seizure manifestation. This is in line with previous reports [49, 51, 52] and underscores the likely preventive role of early and effective ASMs. Severe early-onset epileptic encephalopathies, such as infantile spasms and LGS, were associated with poorer cognitive ability in our patients than focal seizures, as described by others [30, 35, 49, 52, 53]. Moreover, severe intellectual disability was less common in patients with seizure freedom > 1 year at the time of the last visit than in patients with ongoing refractory seizures. This is in line with retrospective data of Nabbout et al. [30]. Similarly, a retrospective study of adult patients with TSC observed a significant impact of the duration of the drug-resistant course of the epilepsy on long-term intellectual ability as well as risk of psychiatric disorders [54]. Furthermore, Overwater et al. [55] described a close relationship between poor neurodevelopmental outcome and higher numbers of ASMs, use of vigabatrin as first or second line medication, and corticosteroid treatment, while better cognitive ability was associated with response to the first ASM.

ASDs are highly prevalent in TSC, especially in TSC2 variants, with reported rates between 14.6 and 21.8% [2, 5]. The low prevalence (11.4%) of ASDs in our study group might be due to missing standardized screening tests; however, the comparability of published studies is limited because of differences in the age and cognitive performance status of the study groups and in variable diagnostic criteria [2]. Behavioral problems, such as hyperactivity, anxiety, and sleep disorders, were not analyzed in the present study because of incomplete data. In the TOSCA PASS [5], in the pediatric age group (< 18 years of age), ASDs were diagnosed in 42.1% and were the most common neuropsychiatric disorders, with the highest prevalence in 2- to 9-year-old children (55.6%), followed by ADHD, depressive disorder, and anxiety disorder. Moavero et al. [56] reviewed prospective systematic data from the EPISTOP study and proposed that a normal cognitive developmental quotient at 12 months of age excluded subsequent ASDs, and that total ADOS score at 12 months and atypical socio-communication behaviors at 24 months may predict a future diagnosis of ASD. While animal studies indicate the positive effects of mTOR inhibitors on neurocognitive abilities [57], these potential effects have yet to be investigated in humans [56, 58]. Considering the high burden of disease due to the various neuropsychiatric disorders, even in the pediatric age group, recommendations of the International TSC Consensus Panel [16] emphasize the significance of early and appropriate assessment of TAND to enable individual early-intervention programs, pharmacological treatments, and support of caregivers [29]. The need for developmentally supportive care among all age groups is underscored by the present data.

### Limitations of the study

This study had some limitations due to its retrospective design and small sample size compared with other studies, which may have limited our conclusions on genotype–phenotype correlations. Missing genetic testing results reduced the statistical power. Because of the incomplete data, we excluded dermatological manifestations and behavioral problems from our analysis.

Further prospective studies on cognitive performance and behavioral problems in defined age groups are necessary to confirm our observations. As this monocenter data sampling included exclusively patients of the local Social Pediatric Centre and the Epilepsy Centre Erlangen with a high ratio of refractory epilepsy, a selection bias cannot be excluded [38]. However, the epilepsy characteristics reported in the present study are in line with comprehensive international studies [6, 30], and we believe that our findings have implications for clinical practice and optimization of multidisciplinary long-term care of TSC patients.

## Conclusions

The present study emphasizes the phenotypic variability of pediatric-onset TSC and high rate of neurological and neuropsychiatric morbidity, including epilepsy, intellectual disability, and ASDs. Early-onset refractory epilepsy is indicated as a relevant prognostic factor for impaired cognitive development. Multidisciplinary management and comprehensive care should be provided for children, adolescents, and adults with TSC in addition to psychosocial support for parents and caregivers.

### Supplementary Information

**Additional file 1: Table A1.** Characteristics of patients < /= 42 months of age in relation to genotype and developmental outcome, n = 9 (age mean ± SD: 2.0 ± 0.8 years). **Table A2.** Characteristics of patients > 42 months of age in relation to genotype and developmental outcome, n = 12 (age, mean ± SD: 11.5 ± 6.8 years).

## Data Availability

All data sets generated during and/or analyzed during the present study are not publicly available but are available from the corresponding author on reasonable request.

## Abbreviations

NMI: No mutation identified
TSC: Tuberous sclerosis complex
TOSCA: TuberOus SClerosis registry to increase disease Awareness
