# Supplementary material for: A single-center observational study on long-term neurodevelopmental outcomes in children with tuberous sclerosis complex
Source: Orphanet J Rare Dis. 2023 Nov 9;18:349. doi: 10.1186/s13023-023-02959-0 (PMC10637019; doi:10.1186/s13023-023-02959-0)
Supplement: Supplementary file 1 — Additional file 1: Table A1. Characteristics of patients < /= 42 months of age in relation to genotype and developmental outcome, n = 9 (age mean ± SD: 2.0 ± 0.8 years). Table A2. Characteristics of patients > 42 months of age in relation to genotype and developmental outcome, n = 12 (age, mean ± SD: 11.5 ± 6.8 years). [file 13023_2023_2959_MOESM1_ESM.docx]

**Supplementary Table A1:** Characteristics of patients </=42 months of age in relation to genotype and developmental outcome, n=9 (age mean±SD: 2.0±0.8 years)

| **Cognitive ability** | **Average**  n (%) | **Below average**  n (%) | **Subgroup:**  **Severe cognitive impairment**  n (%) |
| --- | --- | --- | --- |
| **Genotype** |  |  |  |
| TSC1 |  | 1 | 0 |
| TSC2 | 3/8 (37.5) | 5/8 (62.5) | 2/8 (25.0) |
| **Epilepsy** |  |  |  |
| Age at manifestation (years), mean±SD | (0.5; 1.5) | 0.39 ± 0.36 | (1st week of life;  1.2 months) |
| No epilepsy | 1 | 0 | 0 |
| Epilepsy with focal seizures | 1/5 (20.0) | 4/5 (80.0) | 1/5 (20.0) |
| Multifocal epilepsy with various seizures types (incl. West syndrome) | 1/3 (33.3) | 2/3 (66.7) | 1 (33.3) |
| **Seizure freedom (last visit)** |  |  |  |
| No seizure freedom (last visit) | 2/6 (33.3) | 4/6 (66.7) | 2/6 (33.3) |
| Seizure freedom > 1 year (last visit) | 1/3 | 2/3 | 0 |

**Supplementary Table A2:** Characteristics of patients > 42 months of age in relation to genotype and developmental outcome, n=12 (age, mean±SD: 11.5 ± 6.8 years)

| **Cognitive ability** | **Average**  n (%) | **Below average**  n (%) | **Subgroup:**  **Severe cognitive impairment**  n (%) |
| --- | --- | --- | --- |
| **Genotype** |  |  |  |
| TSC1 | 2/5 | 3/5 | 2/5 |
| TSC2 | 2/7 | 5/7 | 2/7 |
| **Epilepsy** |  |  |  |
| Age at manifestation (years), mean±SD | 5.08 ± 4.88 | 0.56 ± 0.74 | 0.28 ± 0.20 |
| No epilepsy | 1/3 | 2/3 | 0 |
| Epilepsy with focal seizures | 3/6 | 3/6 | 1/6 |
| Multifocal epilepsy with various seizures types (incl. West syndrome) | 0 | 3/3 | 3/3 |
| **Seizure freedom (last visit)** |  |  |  |
| No seizure freedom (last visit) | 2/6 | 4/6 | 1/6 |
| Seizure freedom > 1 year (last visit) | 1/3 | 2/3 | 2/3 |
